# From strain to strength: a yearlong study on the transformative influence of inner engineering online program on mental well-being

**DOI:** 10.3389/fpsyg.2025.1436910

**Published:** 2025-02-04

**Authors:** Ashwin Swaminathan, Braiden DeSchryver, Akila Rayapuraju, Julianna Barbaro, Hibiki Orui, Balachundhar Subramaniam, Preeti Upadhyay Reed

**Affiliations:** Sadhguru Center for a Conscious Planet, Beth Israel Deaconess Medical Center, Harvard Medical School, Boston, MA, United States

**Keywords:** mindfulness, beginner meditation, meditation techniques, inner engineering, yoga, mental health, wellbeing

## Abstract

**Introduction:**

Health is a multidimensional phenomenon encompassing physical, mental, and social well-being, all of which are deeply interconnected. The COVID-19 pandemic highlighted the importance of mental and social health, as rates of loneliness, depression, and anxiety surged. Mindfulness practices, such as Shambhavi Mahamudra Kriya (SMK), have gained attention for their potential to enhance well-being by integrating breath regulation, meditation, and cognitive reframing techniques. During the COVID-19 pandemic, the Inner Engineering Completion Online (IECO) program was created to effectively teach SMK with global travel restrictions in effect. This study examines the long-term effects of SMK, taught through the IECO, on various measures of well-being over a one-year follow-up period.

**Methods:**

Participants were recruited from the January 2020 IECO course. Participants completed surveys at consent, post-IECO, and 6 weeks, 6 months, and 1-year post-IECO. The surveys consisted of 4 validated neuropsychological scales: Perceived Stress Scale (PSS), Positive Emotion/Relationship/Engagement Scale (PERMA) Profiler, Pittsburgh Sleep Quality Index (PSQI), and Mindful Attention Awareness Scale (MAAS). Survey data was analyzed using linear mixed effect modeling. Two-sided *p*-values of <0.05 were considered statistically significant.

**Results:**

Hundred and eighty-eight participants were enrolled. Hundred and sixty-four participants completed baseline measurements, and 41 participants completed surveys at all timepoints. The baseline median [IQR] PSS score in participants was 13 [8, 18]; post-IECO median [IQR] PSS was 11 [8, 16] and 6-week median [IQR] PSS was 7 [4, 12], suggesting that consistent practice of Shambhavi Mahamudra Kriya resulted in reduced stress. This score was sustained up to a year post-IECO with a median [IQR] of 7 [3, 12]. The mean mindfulness scale (MAAS) score increased by 0.97 (95% C.I. 0.7–1.2 *p* < 0.01, η^2^_p_ = 0.30) at the 1-year timepoint compared to baseline. The global PSQI score reduced at the week 6 timepoint by 1.3 (95% C.I. 0.49–2.0, *p* < 0.01) with medium effect size and was sustained until 1 year.

**Discussion:**

Within 6 weeks of participating in IECO, regular practice of SMK significantly reduced stress, improved sleep quality, and boosted mindfulness. These benefits were sustained for at least a year with continued practice, suggesting that this practice is an effective path to maintaining general well-being.

**Clinical trial registration:**

ClinicalTrials.gov, trial identification number NCT04189146.

## Introduction

Health is not, and never has been, a unidimensional phenomenon. In a state of high-quality health, one experiences physical, mental, and social well-being — not solely the absence of disease (Constitution of the World Health Organization, 1948). Physical health is often the primary point of enquiry in many research studies due to its measurable and objective nature. However, mental and social health are equally important contributors to one’s overall well-being. It is necessary to think of these three aspects of health as interconnected entities; each one influences the other. For instance, psychosocial phenomena such as loneliness and social isolation have been shown to be positively correlated with the risk for cardiovascular events (Bu et al., 2020).

In recent years, mindfulness has gained significant public attention due to its widely reported benefits on well-being. Research suggests that mindfulness practices can reduce stress, improve mental health, enhance emotional regulation, foster self-awareness, boost concentration, support physical health, and strengthen relationships (Vago and Silbersweig, 2012; Keng et al., 2011). By cultivating presence, self-awareness, and acceptance, mindfulness positively influences mental, physical and social aspects of well-being. Mental and social well-being gained unprecedented attention during the COVID-19 pandemic. During this period, rates of loneliness, depression, anxiety, unemployment, and suicidality surged (Einav and Margalit, 2023; Pathirathna et al., 2022), creating a mental health crisis that placed immense strain on both the healthcare system and its providers.

It was during this time that the study team examined the effects of Shambhavi Mahamudra Kriya (SMK), an ancient yogic practice incorporating breath regulation, meditation, and mudras (hand gestures) on interested individuals with no prior mindfulness experience. The authors investigated SMK due to its standardized structure and easy accessibility during lockdown, courtesy of its online teaching format.

SMK is a core component of the Inner Engineering Completion Online (IECO) program offered by the Isha Foundation. Inner Engineering is a term that was created by Sadhguru to describe the steps necessary to engineer a more peaceful and joyous life experience. The IECO program integrates yoga practices, philosophical teachings, and SMK into a structured, multimodal framework. The program is broken down into 7 modules. During the first six modules, participants watch videos where they learn cognitive reappraisal techniques, Upa Yoga (pre-yoga), and listen to wisdom talks. In the final module, participants join a live online session where they actively engage in both Upa Yoga and learn SMK all with the guidance of trained Hatha Yoga instructors. Simply put, SMK is a multimodal intervention that combines a crash course on inner engineering (*acceptance, responsibility, seeing oneself as the mother to the world, understanding one’s true self is beyond body and mind*) along with alternative nostril breathing, chanting, rapid breathing, breath holds, and breath watching. SMK is consistent with the NCCIH’s recommendation to use multicomponent methods (Baumgartner, 2024); offering an opportunity for a complete overhaul of mind, body, energy, and emotions. After attending the IECO program, participants are encouraged to practice SMK twice daily for 40 days, followed by once daily thereafter.

Although there is no dearth of literature on the benefits of mind–body interventions, most studies tend to focus on outcomes that are measured within 6 weeks to 6 months of intervention practice (Goyal et al., 2014; Spijkerman et al., 2016). This study is unique in that it investigates the sustained impact of SMK and IECO on various measures of wellbeing, through a year-long follow-up. By increasing the follow-up duration to a year after the practice was taught, this study looks to capture the impact and feasibility of these practices in the long-term. It is hypothesized that regular practice of SMK will result in sustained stress reduction and increase in overall well-being among other results over a year.

## Methods

### Participants and procedure

This study was designed to comply with the institutional review board’s (IRB) requirements at Beth Israel Deaconess Medical Center (BIDMC). The study was initially approved on March 8, 2019, by the IRB at BIDMC and given the protocol number 2019P000205. The manuscript detailing this cohort’s short-term outcomes (i.e., up to 6 weeks from practice initiation) has been previously published elsewhere (Upadhyay et al., 2022). To avoid repetition, a summary of methods is detailed below.

### Recruitment and intervention

In this prospective observational study, participants were recruited through measures such as social media posts, websites, flyers, word of mouth, and email announcements from the Isha Foundation. The study team only recruited participants who were registered to attend the Inner Engineering Completion Online (IECO) course in January 2020, wherein the participants learned a 21-min mindfulness practice called Shambhavi Mahamudra Kriya (SMK). Participants paid their registration fees for the course and no remuneration was offered for participating in this study.

The primary intervention for this study was SMK. SMK is a meditation practice that consists of five unique breath manipulation techniques that are done in a specific order, along with aum chanting and the engagement of bandhas (muscular locks in the abdomen and pelvic floor). The practice concludes with about 5 min of breath-watching meditation. Following the IECO program, participants are asked to meditate twice a day for 40 days and once a day thereafter.

### Data collection and measures

Electronic surveys were completed by all enrolled participants at five key timepoints: baseline, immediate post-intervention, 6 weeks, 6 months, and 1 year after IECO completion. All surveys were collected through the HIPAA-compliant, secure electronic data capture platform REDCap.

**Figure 1:**
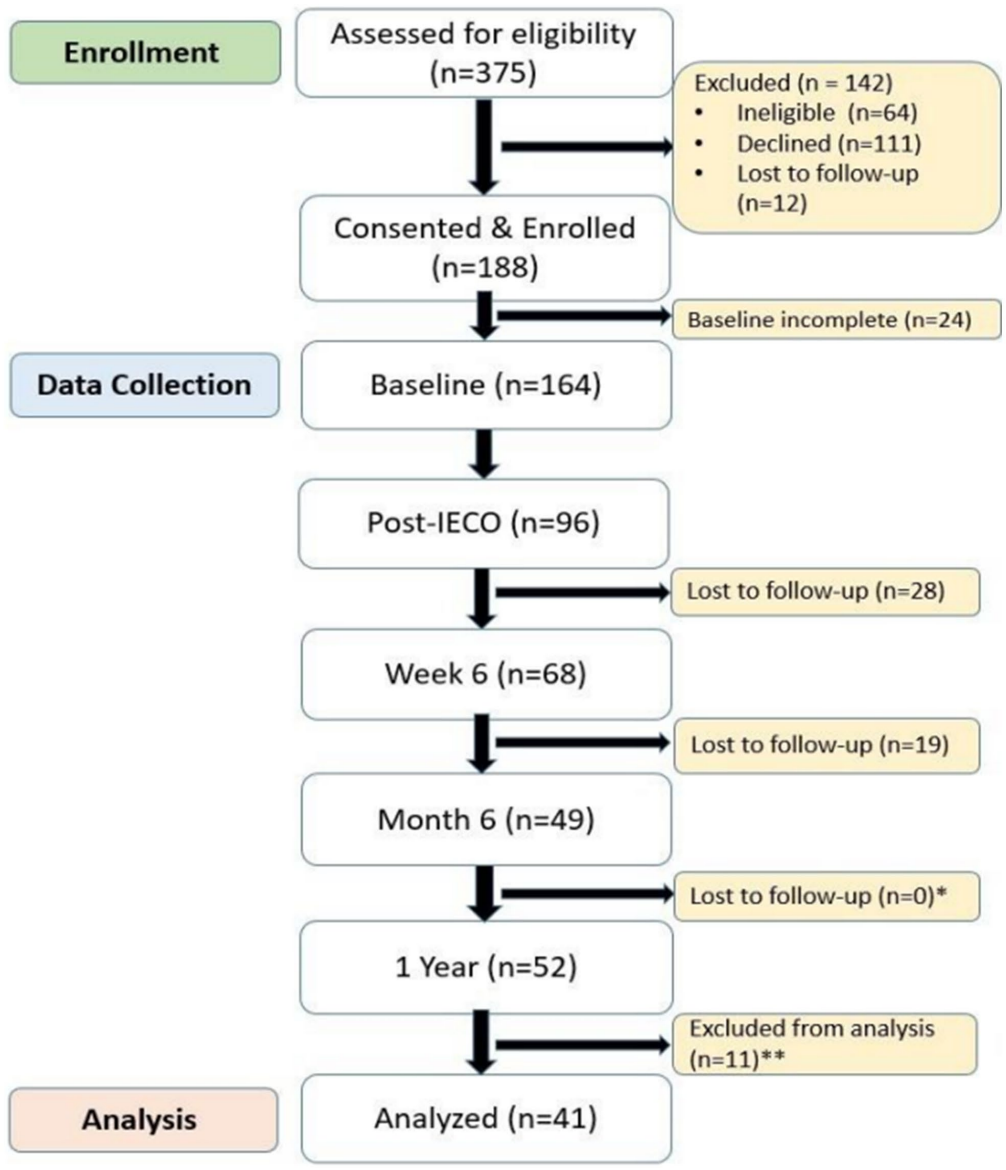
The CONSORT diagram illustrating participant flow for the long-term outcomes of the cohort, adapted from the previous publication showing short-term outcomes (Upadhyay et al., 2022). *Increase in participants between Month 6 and 1 Year is due to participants who completed 1 Year, but not Month 6. **Participants excluded from analysis due to incomplete PSQI data.

Participants’ self-reported responses on their health and other well-being measures were collected through REDCap surveys. Namely, these surveys inquired about participants’ medical history, diet & exercise, habits, history of addiction or substance abuse (smoking/alcohol consumption), etc. Four validated neuropsychological scales were also employed: Perceived Stress Score, PERMA Profiler, Pittsburgh Sleep Quality Index and Mindful Attention Awareness Scale. Further activity diaries were collected from study participants to gain insights on their compliance to the SMK practice. Intervention compliance was defined as 60% of activity completion during the intervention period, which translates to 4 or more days of meditation practice in a week (Figure 1).

### Outcome measures

#### Stress

The 10-item Perceived Stress Scale (PSS) was employed to measure perceived stress levels during this period (Cohen et al., 1983). Each item was coded as 0 “never” to 4 “very often.” The PSS score ranges from 0 to 40, with higher values, suggesting higher stress levels. The reliability of PSS across the study duration was calculated as 0.89 at baseline; 0.91 at post-IECO, 0.91 at week 6, 0.92 at month 6, and 0.93 at 1 year.

#### Mindfulness

The 5-item Mindful Attention Awareness Scale (MAAS) was employed to capture the state effect of Mindfulness (Brown and Ryan, 2003). Each item is coded 1 “never” to 5 “all of the time,” higher values correlating to higher experience of current mindfulness. The reliability of MAAS short scale across the study duration was calculated as 0.89 at baseline; 0.92 at post-IECO, 0.89 at week 6, 0.89 at month 6, and 0.85 at 1 year.

#### PERMA

PERMA a 23-item measure that assesses well-being across five domains (positive emotion, engagement, relationships, meaning, accomplishment) (Butler and Kern, 2016). Questions are reported on an 11-point scale ranging from 0 to 10, with the end points labeled. Scores are calculated as the average of the items comprising each factor with higher scores indicating a greater level of well-being. The reliability of the PERMA scale across the study duration was calculated as 0.67 at baseline, 0.70 at post-IECO, 0.70 at week 6, 0.70 at month 6, and 0.71 at 1 year.

#### Pittsburgh sleep quality index

The PSQI is a 24-item scale that measures sleep disturbances along 7 dimensions: subjective sleep quality, sleep latency, sleep duration, habitual sleep efficiency, sleep disturbances, use of sleep medication, and daytime dysfunction. The PSQI evaluates sleep quality through seven components, each rated from 0 (no difficulty) to 3 (severe difficulty). These scores are combined into a global score ranging from 0 to 21, with higher scores reflecting poorer sleep quality. Responses are based on most days (and nights) of the previous month (Buysse et al., 1989). The reliability of PSQI global score across study duration was calculated to be 0.55 at baseline; 0.35 at post-IECO and 0.54 at week 6, 0.29 at month 6, and 0.58 at 1 year.

### Statistical analysis

Descriptive statistics of the data were presented based on the types of variables and distribution. Continuous data were presented as medians and interquartile ranges (IQR) for non-normal distributed data. Categorical data were presented as frequencies and proportions. Unadjusted repeated measure analyses were performed using Nonparametric Friedman one-way repeated measure analysis test or one-way ANOVA test with repeated measures to observe overall changes throughout the study period. Adjusted repeated measure analyses were performed using multivariable linear mixed effect models to measure differences between timepoints controlling for possible cofounders. Statistical analyses were conducted using R (Version 4.2.3) with two-sided hypotheses testing and *p*-values <0.05 were considered statistically significant. Kendall’s coefficient of concordance (W) from nonparametric analyses and partial eta square (η^2^_p_) from one-way ANOVA and linear mixed effect models were computed as effect size.

## Results

### Baseline characteristics

Table 1 shows baseline demographics, which includes age, gender, race and ethnicity, educational qualifications, and employment status. Of the 41 participants that completed the 1-year follow-up, the sample is predominantly white (54%) and female (59%). This sample was also well-educated, with 56% of the sample having at least completed a bachelor’s degree and 61% of the sample being employed full-time. Contrary to popular belief that MBI practices cater to those who are either retirees or those with ample time to spend. The study cohort revealed that those who completed this year-long study were gainfully employed, well-educated and predominantly in their midlife, underscoring the accessibility and relevance of these practices in their lives.

**Table 1:** Demographic profile.

	*N* (%)
Sex, No. (%)	
Female	24 (59)
Male	16 (39)
I prefer not to specify	1 (2)
Age (years), No. (%)	
18–29	7 (17)
30–39	4 (10)
40–49	8 (20)
50–59	12 (29)
60 +	9 (22)
70 +	1 (2)
Race, No. (%)	
White	22 (54)
Black or African American	0
Asian	6 (15)
Native Hawaiian or Other Pacific Islander	1 (2)
American Indian or Alaskan Native	0
Multi-Racial	5 (12)
Other	5 (12)
Ethnicity, No. (%)	
Hispanic/Latino	11 (27)
Not Hispanic/Latino	27 (66)
Prefer not to specify	3 (7)
Educational qualifications, No. (%)	
High School/GED	3 (7)
Some college, Associate’s Degree	15 (37)
Undergraduate Degree (Bachelor’s)	13 (32)
Graduate Degree (Master’s)	9 (22)
Doctoral Degree (Ph.D. or equivalent degree)	1 (2)
Employment status, No. (%)	
Unemployed	8 (20)
Employed: Part-Time	6 (15)
Employed: Full-Time	25 (61)
I prefer not to specify	2 (5)

### Primary outcome—perceived stress score

**Figure 2:**
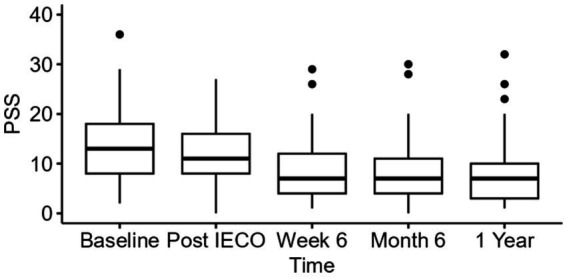
Time trend comparison of PSS scores at all three timepoints. The figure displays median (IQR) for PSS scores of all time points (*n* = 41) until 1 year period.

Nonparametric Friedman one-way repeated measure analysis showed significant effect (*p* < 0.01) with small to moderate effect size (Kendall’s *W* = 0.27 for 1 year) on reducing for PSS scores. As an adjusted repeated measure analysis using linear mixed effect model controlling age, gender, race, highest education level, and employment status, it was found that mean PSS score reduction of 5.1 (95% C.I. 3.5–6.8, *p* < 0.01, η^2^_p_ = 0.29) at the 1-year timepoint compared to baseline with a large effect size (Tables 2, 3). PSS score was greatly reduced at the week 6 timepoint, with a drop of 4.7 (95% C.I. 3.1–6.3, *p* < 0.01) from baseline, then sustained its level until the 1-year timepoint (Figure 2).

**Table 2:** PSS scores—primary outcome (primary outcome comparison at four timepoints).

	Baseline	Post IEOC	Week 6	Month 6	1 Year
PSS	13 (8, 18)	11 (8, 16)	7 (4, 12)	7 (4, 11)	7 (3, 10)

**Table 3:** PSS Score—adjusted model.

	Beta	95% CI*^1^*	p-value
Time			
Baseline	—	—	
Post IECO	−1.6	−3.2, 0.01	0.05
Week 6	−4.7	−6.3, −3.1	<0.01
Month 6	−5.0	−6.6, −3.4	<0.01
1 Year	−5.1	−6.8, −3.5	<0.01
Age			
18–29	—	—	
30–39	−4.7	−15, 5.6	0.4
40–49	−5.3	−15, 4.2	0.3
50–59	−5.9	−18, 6.1	0.3
60–69	−9.6	−20, 1.2	0.08
70+	−14	−31, 2.9	0.10
Gender			
Male	—	—	
Female	−0.39	−6.8, 6.0	0.9
I prefer not to specify	−1.7	−18, 14	0.8
Race			
White	—	—	
Asian	0.63	−9.3, 11	0.9
Multi-Race	−4.4	−12, 3.7	0.3
Native Hawaiian	−0.78	−15, 14	>0.9
Other	−0.07	−8.8, 8.6	>0.9
Unknown	−1.4	−15, 12	0.8
Education			
Master’s	—	—	
Associate’s	−0.96	−9.0, 7.1	0.8
Bachelor’s	2.8	−4.6, 10	0.4
High School	−0.44	−11, 10	>0.9
Employment			
Full-Time	—	—	
Part-Time	1.1	−6.4, 8.6	0.8
Unemployed	−1.7	−8.4, 5.0	0.6
Unknown	−0.05	−11, 11	>0.9

^1CI, confidence interval.^

### Secondary outcomes

#### Mindfulness scale (MAAS)

A one-way repeated measure analysis was performed to measure MAAS score increase over the 1-year period. The unadjusted analysis showed significant effect (*p* < 0.01) on increasing for MAAS scores with large effect size (η^2^_p_ = 0.21 for 6 months, η^2^_p_ = 0.32 for 1 year) throughout the practice period.

**Figure 3:**
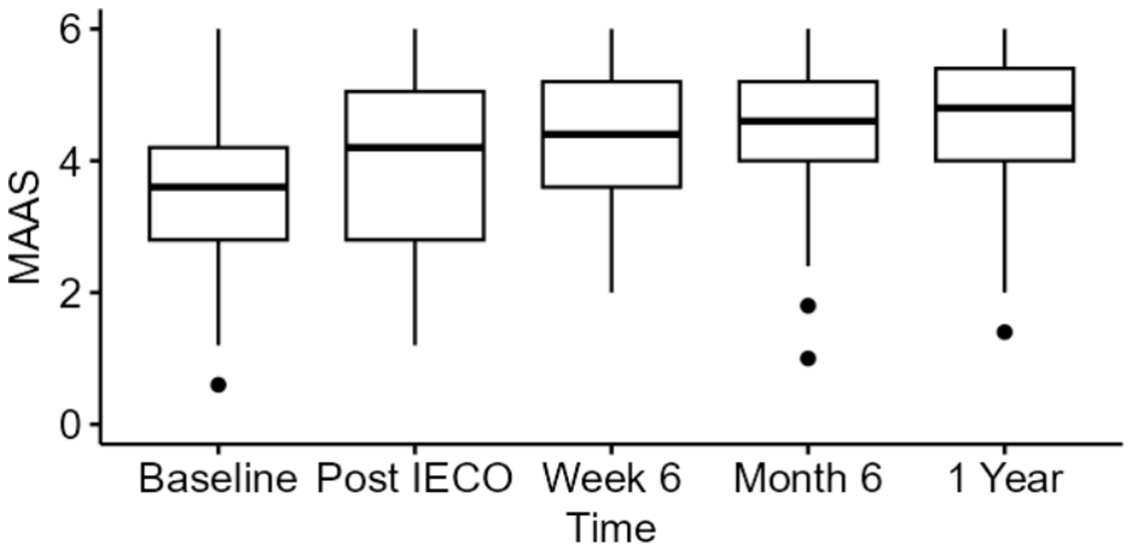
Time trend comparison of MAAS scores at all three timepoints. The figure displays median (IQR) for PSS scores of all time points until 1 year period.

When applying adjusted repeated measure analyses using linear mixed effect models controlling for age, gender, race, highest education level, and employment status, a mean MAAS score increase by 0.97 (95% C.I. 0.7–1.2 *p* < 0.01, η^2^_p_ = 0.30) at the 1-year timepoint compared to baseline with a large effect size was found (Tables 4, 5 and Figure 3).

**Table 4:** MAAS scores—secondary outcome (comparison 5 timepoints).

	Baseline	Post IECO	Week 6	Month 6	1 Year	*p*-value
MAAS	3.6 (2.8, 4.2)	4.2 (2.8, 5.1)	4.4 (3.6, 5.2)	4.6(4.0, 5.2)	4.8 (4.0, 5.4)	<0.01

**Table 5:** MAAS adjusted analysis.

	Beta	95% CI^1^	*p*-value
Time			
Baseline	—	—	
Post IECO	0.36	0.08, 0.63	0.01
Week 6	0.75	0.48, 1.0	<0.01
Month 6	0.85	0.58, 1.1	<0.01
1 Year	0.97	0.70, 1.2	<0.01
Age			
18–29	—	—	
30–39	0.22	−1.4, 1.8	0.8
40–49	0.57	−0.90, 2.0	0.4
50–59	0.92	−0.93, 2.8	0.3
60–69	0.97	−0.70, 2.6	0.2
70+	3.4	0.72, 6.0	0.02
Gender			
Male	—	—	
Female	0.26	−0.73, 1.3	0.6
I prefer not to specify	0.43	−2.0, 2.9	0.7
Race			
White	—	—	
Asian	0.09	−1.4, 1.6	>0.9
Multi-Race	0.49	−0.77, 1.7	0.4
Native Hawaiian	1.1	−1.1, 3.4	0.3
Other	−0.14	−1.5, 1.2	0.8
Unknown	0.66	−1.4, 2.7	0.5
Education			
Master’s	—	—	
Associate’s	0.35	−0.89, 1.6	0.6
Bachelor’s	−0.28	−1.4, 0.85	0.6
High School	0.76	−0.92, 2.4	0.4
Employment			
Full-Time	—	—	
Part-Time	−1.1	−2.2, 0.08	0.07
Unemployed	0.18	−0.86, 1.2	0.7
Unknown	−0.04	−1.7, 1.7	>0.9

^1CI, confidence interval.^

#### Positive emotion/relationship/engagement scale (PERMA)

Non-normal distributions were observed for each timepoint on PERMA subscales. Thus, nonparametric Friedman one-way repeated measure analysis was performed to measure change in each of the PERMA subscales over the year. The unadjusted analysis showed significant effect (*p* < 0.01) on changing scores with small effect size on subscales throughout the practice period.

**Figure 4:**
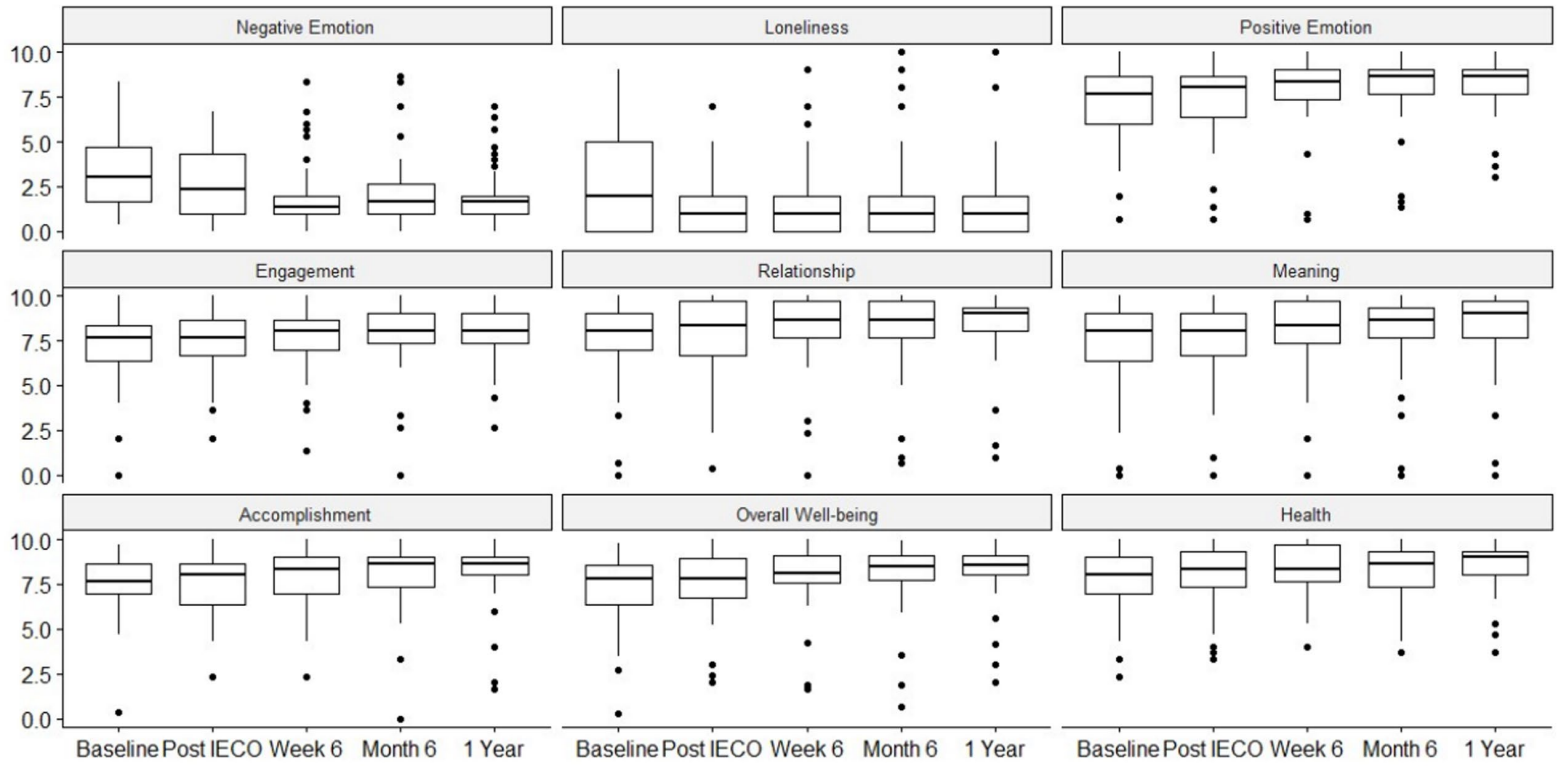
Time trend comparison of all PERMA subscales for five time points.

When applying an adjusted repeated measure analysis using linear mixed effect model controlling for age, gender, race, highest education level, and employment status, it was found that most of the subscales except Loneliness and Health showed significant reduction at the 6-month timepoint (Tables 6, 7). Small effect sizes were observed in the Loneliness subscale, medium effect sizes were observed in the Engagement, Relationship, Meaning, and Accomplishment subscales, and large effect sizes were observed in the Negative Emotion, Positive Emotion, Overall Well-being, and Health subscales (Figure 4).

**Table 6:** PERMA scores—secondary outcome (comparison at five timepoints).

	Baseline^1^	Post IECO^1^	Week 6^1^	Month 6^1^	1 Year^1^	*p*-value
PERMA scale, negative affect measures						
Negative emotion	3.0 (1.7, 4.7)	2.3 (1.0, 4.3)	1.3 (1.0, 2.0)	1.7 (1.0, 2.7)	1.7 (1.0, 2.0)	<0.01
Loneliness	2.0 (0.0, 5.0)	1.0 (0.0, 2.0)	1.0 (0.0, 2.0)	1.0 (0.0, 2.0)	1.0 (0.0, 2.0)	<0.01
PERMA scale, positive affect measures						
Positive emotion	7.7 (6.0, 8.7)	8.0 (6.3, 8.7)	8.3 (7.3, 9.0)	8.7 (7.7, 9.0)	8.7 (7.7, 9.0)	<0.01
Engagement	7.7 (6.3, 8.3)	7.7 (6.7, 8.7)	8.0 (7.0, 8.7)	8.0 (7.3, 9.0)	8.0 (7.3, 9.0)	0.13
Relationship	8.0 (7.0, 9.0)	8.3 (6.7, 9.7)	8.7 (7.7, 9.7)	8.7 (7.7, 9.7)	9.0 (8.0, 9.3)	<0.01
Meaning	8.0 (6.3, 9.0)	8.0 (6.7, 9.0)	8.3 (7.3, 9.7)	8.7 (7.7, 9.3)	9.0 (7.7, 9.7)	<0.01
Accomplishment	7.7 (7.0, 8.7)	8.0 (6.3, 8.7)	8.3 (7.0, 9.0)	8.7 (7.3, 9.0)	8.7 (8.0, 9.0)	<0.01
Overall well-being	7.8 (6.4, 8.6)	7.8 (6.8, 8.9)	8.1 (7.6, 9.1)	8.5 (7.8, 9.1)	8.6 (8.0, 9.1)	<0.01
Health	8.0 (7.0, 9.0)	8.3 (7.3, 9.3)	8.3 (7.7, 9.7)	8.7 (7.3, 9.3)	9.0 (8.0, 9.3)	<0.01

^1^Median (IQR).

**Table 7:** Changes from baseline using adjusted models for all PERMA subscale outcomes.

	Change from baseline with 95% CI		*p*-value*	Effect size η^2^_p_
PERMA scale, negative affect measures				
Negative emotion				0.21
Post IECO	−0.44	−0.90, 0.02	0.06	
Week 6	−1.2	−1.6, −0.70	<0.001	
Month 6	−1.0	−1.5, −0.55	<0.001	
1 Year	−1.3	−1.7, −0.82	<0.001	
Loneliness				0.06
Post IECO	−0.95	−1.8, −0.08	0.03	
Week 6	−1.0	−1.9, −0.13	0.03	
Month 6	−0.61	−1.5, 0.26	0.2	
1 Year	−1.4	−2.3, −0.52	<0.01	
PERMA scale, positive affect measures				
Positive Emotion				0.19
Post IECO	0.09	−0.32, 0.50	0.7	
Week 6	0.70	0.29, 1.1	0.001	
Month 6	0.81	0.40, 1.2	<0.01	
1 Year	1.0	0.62, 1.4	<0.01	
Engagement				0.10
Post IECO	0.27	−0.16, 0.70	0.2	
Week 6	0.59	0.16, 1.0	<0.01	
Month 6	0.63	0.20, 1.1	<0.01	
1 Year	0.79	0.36, 1.2	<0.01	
Relationship				0.15
Post IECO	0.33	−0.02, 0.67	0.07	
Week 6	0.73	0.38, 1.1	<0.01	
Month 6	0.59	0.24, 0.93	<0.01	
1 Year	0.80	0.45, 1.1	<0.01	
Meaning				0.13
Post IECO	0.14	−0.32, 0.59	0.5	
Week 6	0.74	0.28, 1.2	<0.01	
Month 6	0.64	0.18, 1.1	<0.01	
1 Year	0.91	0.46, 1.4	<0.01	
Accomplishment				0.09
Post IECO	0.07	−0.30, 0.43	0.7	
Week 6	0.42	0.05, 0.79	0.03	
Month 6	0.50	0.13, 0.86	<0.01	
1 Year	0.60	0.23, 0.97	<0.01	
Overall well being				0.20
Post IECO	0.18	−0.13, 0.50	0.3	
Week 6	0.64	0.33, 0.96	<0.01	
Month 6	0.65	0.33, 0.96	<0.01	
1 Year	0.83	0.52, 1.2	<0.01	
Health				0.17
Post IECO	0.14	−0.20, 0.48	0.4	
Week 6	0.64	0.30, 0.98	<0.01	
Month 6	0.31	0.07, 0.76	0.02	
1 Year	0.85	0.50, 1.2	<0.01	

#### Pittsburgh sleep quality index (PSQI)

One-way repeated measure analysis were performed to measure Global PSQI score reduction over the year. The unadjusted analysis showed significant effect (*p* < 0.01) on reducing for PSQI scores with medium effect size (η^2^_p_ = 0.09 for 6 months, η^2^_p_ = 0.12 for 1 year) throughout the practice period.

When applying adjusted repeated measure analyses using linear mixed effect models controlling for age, gender, race, highest education level, and employment status, it was found that a mean Global PSQI score reduction by 1.1 (95% C.I. 0.3–1.9 *p* < 0.01, η^2^_p_ = 0.12) at the 1-year timepoint compared to baseline with medium effect size (Tables 8, 9).

**Table 8:** Global PSQI Scores – secondary outcome (comparison at four timepoints).

	Baseline	Post IECO	Week 6	Month 6	1 Year	*p*-value^2^	Effect size η^2^_p_
PSQI	5.0 (4.0, 8.0)	5.0 (4.0, 7.0)	4.0 (3.0, 5.0)	4.0 (3.0, 6.0)	4.0 (2.0, 6.0)	<0.01	0.12

**Table 9:** PSQI adjusted model.

Characteristic	Beta	95% CI^1^	*p*-value
Time			
Baseline	—	—	
Post IECO	−0.41	−1.2, 0.36	0.3
Week 6	−1.3	−2.0, −0.49	< 0.01
Month 6	−1.5	−2.3, −0.76	< 0.01
1 Year	−1.1	−1.9, −0.32	< 0.01
Age			
18–29	—	—	
30–39	−2.9	−7.1, 1.2	0.2
40–49	−2.2	−6.1, 1.6	0.2
50–59	−1.9	−6.7, 2.9	0.4
60–69	−1.9	−6.3, 2.4	0.4
70+	−0.51	−7.4, 6.4	0.9
Gender			
Male	—	—	
Female	0.71	−1.9, 3.3	0.6
I prefer not to specify	2.9	−3.5, 9.4	0.4
Race			
White	—	—	
Asian	0.01	−4.0, 4.0	>0.9
Multi-Race	−3.5	−6.8, −0.25	0.04
Native Hawaiian	−1.8	−7.6, 4.1	0.5
Other	−0.42	−3.9, 3.1	0.8
Unknown	−2.8	−8.2, 2.6	0.3
Education			
Master’s	—	—	
Associate’s	1.4	−1.8, 4.6	0.4
Bachelor’s	1.3	−1.6, 4.3	0.4
High School	1.3	−3.1, 5.7	0.5
Employment			
Full-Time	—	—	
Part-Time	−0.10	−3.1, 2.9	>0.9
Unemployed	0.62	−2.1, 3.3	0.6
Unknown	−0.06	−4.5, 4.3	>0.9

^1CI, confidence interval.^

**Figure 5:**
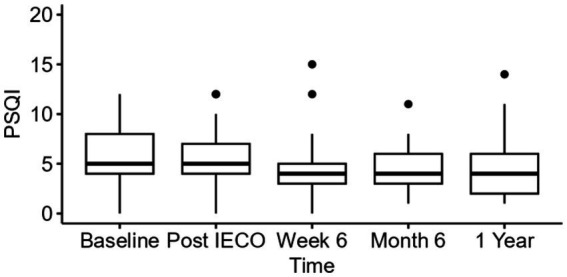
Time trend comparison of Global PSQI scores at all three timepoints. The figure displays median (IQR) for PSQI scores.

It can be concluded Global PSQI score reduced at the week 6 timepoint by 1.3 (95% C.I. 0.49–2.0, *p* < 0.01) with medium effect size, then sustained its level until the 1-year timepoint (Figure 5).

### Pearson correlation among the outcomes

Pearson correlations of changes were computed between baseline to 1-year among all outcomes (Tables 10, 11). Notably, changes in PSS and MAAS had moderate negative correlation (*ρ* = −0.4, *p* < 0.01), indicating that changes in PSS and MAAS showed a significant linear relationship.

**Table 10:** Correlation ρ values among outcome changes (baseline to 1 year).

	PSS	PSQI	MAAS	PERMA Negative Emotion	PERMA Loneliness	PERMA Positive Emotion	PERMA Engagement	PERMA Relationship	PERMA Meaning	PERMA Accomplishment	PERMA Overall wellbeing	PERMA Health
PSS	–											
PSQI	−0.22	–										
MAAS	−0.4	0.17	–									
PERMA negative emotion	0.66	−0.12	−0.5	–								
PERMA loneliness	0.27	−0.04	0.05	0.21	–							
PERMA positive emotion	−0.73	0.1	0.34	−0.66	−0.06	–						
PERMA engagement	−0.27	0.09	0.27	−0.19	0.06	0.48	–					
PERMA relationship	−0.56	0.08	0.36	−0.55	0	0.68	0.64	–				
PERMA meaning	−0.59	0.17	0.34	−0.57	0.07	0.74	0.59	0.61	–			
PERMA accomplishment	−0.59	0.02	0.46	−0.56	−0.09	0.66	0.51	0.55	0.68	–		
PERMA overall wellbeing	−0.66	0.12	0.41	−0.62	−0.02	−0.87	0.77	0.83	0.87	0.81	–	
PERMA health	−0.31	0.13	0.48	−0.45	0.01	0.5	0.69	0.68	0.56	0.53	0.71	–

**Table 11:** Correlation ρ values with *p*-value among outcome changes (baseline to 1 year).

		Correlation	*p*-value
PSS	PSQI	−0.22	0.14
PSS	MAAS	−0.4	<0.01
PSS	PERMA negative emotion	0.66	<0.01
PSS	PERMA loneliness	0.27	0.06
PSS	PERMA positive emotion	−0.73	<0.01
PSS	PERMA engagement	−0.27	0.06
PSS	PERMA relationships	−0.56	<0.01
PSS	PERMA meaning	−0.59	<0.01
PSS	PERMA accomplishment	−0.59	<0.01
PSS	PERMA overall wellbeing	−0.66	< 0.01
PSS	PERMA health	−0.31	0.03
PSQI	MAAS	0.17	0.25
PSQI	PERMA negative emotion	−0.12	0.41
PSQI	PERMA loneliness	−0.041	0.78
PSQI	PERMA positive emotion	0.1	0.48
PSQI	PERMA engagement	0.092	0.53
PSQI	PERMA relationships	0.083	0.57
PSQI	PERMA meaning	0.17	0.25
PSQI	PERMA accomplishment	0.016	0.91
PSQI	PERMA overall wellbeing	0.12	0.43
PSQI	PERMA health	0.13	0.36
MAAS	PERMA negative emotion	−0.5	<0.01
MAAS	PERMA loneliness	0.05	0.72
MAAS	PERMA positive emotion	0.34	0.02
MAAS	PERMA engagement	0.27	0.06
MAAS	PERMA relationships	0.36	0.01
MAAS	PERMA meaning	0.34	0.02
MAAS	PERMA accomplishment	0.46	<0.01
MAAS	PERMA overall wellbeing	0.41	<0.01
MAAS	PERMA health	0.48	<0.01
PERMA negative emotion	PERMA loneliness	0.21	0.16
PERMA negative emotion	PERMA positive emotion	−0.66	<0.01
PERMA negative emotion	PERMA engagement	−0.19	0.18
PERMA negative emotion	PERMA relationships	−0.55	<0.01
PERMA negative emotion	PERMA meaning	−0.57	<0.01
PERMA negative emotion	PERMA accomplishment	−0.56	<0.01
PERMA negative emotion	PERMA overall wellbeing	−0.62	<0.01
PERMA negative emotion	PERMA health	−0.45	<0.01
PERMA loneliness	PERMA positive emotion	−0.058	0.69
PERMA loneliness	PERMA engagement	0.058	0.69
PERMA loneliness	PERMA relationships	−0.0047	0.97
PERMA loneliness	PERMA meaning	0.067	0.65
PERMA loneliness	PERMA accomplishment	−0.093	0.52
PERMA loneliness	PERMA overall wellbeing	−0.022	0.88
PERMA loneliness	PERMA health	0.011	0.94
PERMA positive emotion	PERMA engagement	0.48	<0.01
PERMA positive emotion	PERMA relationships	0.68	<0.01
PERMA positive emotion	PERMA meaning	0.73	<0.01
PERMA positive emotion	PERMA accomplishment	0.66	<0.01
PERMA positive emotion	PERMA overall wellbeing	0.87	<0.01
PERMA positive emotion	PERMA health	0.5	<0.01
PERMA engagement	PERMA relationships	0.64	<0.01
PERMA engagement	PERMA meaning	0.59	<0.01
PERMA engagement	PERMA accomplishment	0.51	<0.01
PERMA engagement	PERMA overall wellbeing	0.77	<0.01
PERMA engagement	PERMA health	0.69	<0.01
PERMA relationships	PERMA meaning	0.61	<0.01
PERMA relationships	PERMA accomplishment	0.55	<0.01
PERMA relationships	PERMA overall wellbeing	0.83	<0.01
PERMA relationships	PERMA health	0.68	<0.01
PERMA meaning	PERMA accomplishment	0.68	<0.01
PERMA meaning	PERMA overall wellbeing	0.87	<0.01
PERMA meaning	PERMA health	0.56	<0.01
PERMA accomplishment	PERMA overall wellbeing	0.81	<0.01
PERMA accomplishment	PERMA health	0.53	<0.01
PERMA overall wellbeing	PERMA health	0.71	<0.01

### Comment on study adherence

Through using multivariate logistic regression, no notable differences in the demographic characteristics of those who completed the 1-year survey when compared to those who did not were found. However, it was interesting to note that those with doctoral degree had lower odds of completing the 1-year survey compared to those with an associate degree [OR = 0.11 (*p* = 0.061)] (Tables 12, 13).

**Table 12:** Demographic tables for 1 year complete and non-completers.

	Complete, *N* = 41	Not completed, *N* = 123
Gender		
Female	24 (59%)	84 (68%)
I prefer not to specify	1 (2.4%)	2 (1.6%)
Male	16 (39%)	37 (30%)
Age		
18–29	7 (17%)	10 (8.1%)
30–39	4 (9.8%)	28 (23%)
40–49	8 (20%)	28 (23%)
50–59	12 (29%)	29 (24%)
60–69	9 (22%)	16 (13%)
70+	1 (2.4%)	10 (8.1%)
Unknown	0 (0%)	2 (1.6%)
Race		
African American	0 (0%)	3 (2.4%)
Asian	6 (15%)	37 (30%)
Multi-Race	5 (12%)	9 (7.3%)
Native Hawaiian	1 (2.4%)	0 (0%)
Other	5 (12%)	11 (8.9%)
Unknown	2 (4.9%)	1 (0.8%)
White	22 (54%)	62 (50%)
Education		
High School	3 (7.3%)	10 (8.1%)
Associate’s	15 (37%)	19 (15%)
Bachelor’s	13 (32%)	40 (33%)
Master’s	9 (22%)	40 (33%)
Doctoral	1 (2.4%)	14 (11%)
Employment		
Full-Time	25 (61%)	60 (49%)
Part-Time	6 (15%)	27 (22%)
Unemployed	8 (20%)	27 (22%)
Unknown	2 (4.9%)	9 (7.3%)

^1^n (%); Median (IQR).

**Table 13:** Logistic regression on study adherence till 1 year period.

	OR^1^	95% CI^1^	*p*-value
Gender			
Female	—	—	
Male	1.68	0.68, 4.18	0.3
I prefer not to specify	2.81	0.09, 95.7	0.5
Age			
18–29	—	—	
30–39	0.22	0.04, 1.09	0.068
40–49	0.37	0.08, 1.72	0.2
50–59	0.58	0.13, 2.59	0.5
60–69	0.89	0.19, 4.23	0.9
70+	0.23	0.01, 2.07	0.2
Unknown	0.00		>0.9
Race			
Asian	—	—	
African American	0.00		>0.9
Multi-Race	2.22	0.44, 11.0	0.3
Native Hawaiian*	NA	NA	>0.9
Other	2.41	0.45, 12.4	0.3
Unknown	5.73	0.35, 165	0.2
White	1.62	0.47, 5.97	0.5
Education			
Associate’s	—	—	
High School	0.30	0.05, 1.36	0.14
Bachelor’s	0.51	0.17, 1.45	0.2
Master’s	0.42	0.12, 1.36	0.2
Doctoral	0.11	0.01, 0.80	0.061
Employment			
Full-Time	—	—	
Part-Time	0.44	0.13, 1.34	0.2
Unemployed	0.64	0.19, 2.02	0.5
Unknown	1.08	0.12, 6.69	>0.9

^1^ OR, Odds ratio; CI, confidence interval. *Unable to compute estimates.

## Discussion

The aim of conducting this study was to assess whether implementation of the IECO intervention wherein participants learn the SMK practice virtually could have positive long-term effects on health and well-being measures. The variables stress, sleep, emotions, and mindfulness were chosen because they are key indicators associated with overall wellbeing and are often impacted by mindfulness-based interventions (Zhang et al., 2021). Stress and emotions are measures of mental health, while sleep quality reflects an aspect of physical and psychological health, all of which are essential for holistic health. Mindfulness was measured as it is a fundamental component of the IECO intervention and is linked to improvements in stress, emotional regulation, and well-being (Gu et al., 2015). Together, these variables provide a comprehensive profile of how participants are influenced by IECO and SMK. This supports the study’s objective of assessing IECO and SMK’s impact on overall well-being. This section identifies and expands on the key findings from this study.

The IECO program had immediate effects on stress; participants’ median perceived stress score decreased from 13 at baseline to 7 by 6 weeks post-program (Table 2 and Figure 2). Scores of 0–13 on the PSS are considered low self-perceived stress, whereas scores of 14–26 are considered moderate stress (Torales et al., 2020). Thus, on average, participants had low to moderate stress levels at baseline, but the intervention helped further reduce their perceived stress more soundly into the “low-stress” regime. This initial reduction in stress seen after IECO can be compared to the stress reduction felt after vacations. Vacations and meditation retreats have also been shown to have similar effects on cellular health and gene expression (Epel et al., 2016). However, it is well-documented that the effect of vacations on stress is fleeting, and stress levels return to baseline within 2 weeks of returning to a daily working routine (de Bloom et al., 2009, 2013). In contrast, participants who underwent IECO exhibited a sustained reduction in stress; median PSS remained at 7 for up to a year post-intervention.

A similarly sustained reduction in stress can be seen in other interventions, such as Mindfulness in Motion (MIM). In this 8-week intervention, participants attend a one-time 2-h “retreat” and attend 1-h group sessions each week. This practice combines yoga-based stretches with relaxing music (Klatt et al., 2015). In comparison, IECO only requires a one-time online initiation for participants. After this, participants practice the 21-min Shambhavi Mahamudra Kriya meditation twice a day at their own convenience and can attend support sessions as needed. The study team believes that the program’s flexibility is a strong advantage of the IECO, enabling participants to tailor their practice to their own schedules and request support if necessary. Notably, IECO caused comparably sustained stress reduction even with its greater flexibility.

Programs like MIM and IECO employ mindfulness as a means to combat stress. The results of this study show that MAAS scores, indicative of mindfulness, gradually improved for a year after IECO (Table 4 and Figure 3). IECO fosters mindfulness by teaching participants to become continually aware of the several internal and external factors that are affecting their emotional state while continually reappraising these factors for a more mindful experience. Through increased awareness, one may become less reactive to these stimuli and find greater peace and joy in their experiences (Sadhguru, 2016). This technique aligns with Mindfulness-Based Stress Reduction (MBSR), a well-established practice inspired by Buddhist meditations that has also been shown to reduce anxiety and boost mindfulness (Goldin and Gross, 2010). Like IEO, MBSR teaches participants to nonjudgmentally pay intentional attention to the experiences unfolding in front of them (Dundas et al., 2016; Kabat-Zinn, 2003). Evidence increasingly suggests that improved mindfulness results in greater self-compassion, which in turn positively impacts well-being (Evans et al., 2018). Thus, the sustained effect on mindfulness that IECO exhibits could be a pathway to increased general well-being.

IECO’s capacity to improve overall well-being is evidenced by the sustained improvements seen in the subscales of the PERMA profiler, namely, reduction in all negative affect measures and increase in all positive affect measures (Table 6 and Figure 4). Prior work on comparable interventions has shown a similarly positive relationship between mindfulness and well-being. For example, a study on an 8-week intervention called Mindfulness-based strengths practice (MBSP) showed an increase in well-being, engagement, meaning, and health after the intervention (Wingert et al., 2022). Another study examining both mindfulness meditation and positive psychology programs demonstrated an increase in well-being, meaning, and lifestyle measures and reduction in negative emotion (Martin et al., 2021). However, both these studies showed that their interventions had effects on some, but not all subscales of the PERMA profiler. With IECO, moderate effect sizes were seen in all subscales, including those that did not improve with interventions like MBSP, such as Accomplishment, Meaning, and Relationships (Table 7). Future research on IECO may be able to elucidate more information about the effects of SMK on domains of participants’ lives such as Accomplishment, Meaning, and Relationships by combining quantitative scales such as PERMA with qualitative research methods.

Overall well-being and positive affect have also been linked to improved sleep quality (Bower et al., 2010; Zhai et al., 2018). Participants reported that their subjective sleep quality stayed the same or slightly improved over the course of a year after IECO (Table 8 and Figure 5). This falls in line with prior research on mindfulness interventions, with a meta-analysis of several studies on Mindfulness Meditation showing an improved PSQI score in meditators compared to controls (Gong et al., 2016). Interestingly, within participants, these improvements in PSQI were not significant. Research on yogic meditation for healthcare professionals has found similar differences in PSQI between meditators and controls, in addition to improvement in polysomnography scores (Guerra et al., 2020). Thus, the slight improvement in sleep quality experience by IECO participants falls in line with prior research on meditation and sleep and may be linked to overall well-being.

The study’s main strength lies in its longitudinal design and incorporation of a wide variety of validated scales. Collecting 6-month and 1-year follow up data provides robust evidence of the lasting effects of IECO on participants; this longitudinal follow-up is often lacking in current literature on mindfulness and meditation programs. Several studies on meditation programs that use surveys to track measures of well-being, such as the ones on MIM, MBSR, and MBSP referenced previously, commonly employ a pre−/post-intervention approach, where participants’ responses are not collected beyond a couple weeks after the intervention. As such, these studies are unable to capture the resiliency of the positive impacts felt by participants after a meditation program. Additionally, such studies commonly focus on one or just a few potential dimensions of change in a participant’s life, such as sleep, stress, or well-being. By simultaneously measuring long-term changes in stress, mindfulness, overall well-being, and subjective sleep quality, this study helps create a comprehensive profile on how participants’ daily lives are impacted after IECO.

A limitation of this study is its lack of randomization. As with any observational study design, this study is limited by potential self-selection and confounding biases. These biases make it difficult to draw robust causal inferences from the correlations observed in the study (Boyko, 2013). The study was also limited by the exclusive utilization of self-reported survey questionnaires, as self-report bias could impact some of the results. Lastly, high attrition rate was a salient limitation of this study. A 72% attrition rate was observed between enrollment and the 1-year timepoint (Figure 1). This is in-line with current literature finding attrition rates of 0–90% in similar studies on mind–body interventions (Jiang et al., 2021). Although at first glance a 72% attrition rate may seem quite high, it is important to consider that the attrition rate between the post-intervention and 1-year post-IECO timepoints was only 46%. Additionally, of those who completed 6 weeks of practice, the attrition rate was only 24%, suggesting that those participants who were open to committing to an MBI were more likely to continue the practice long term. Akin to any behavioral measures, openness to change is an important factor in MBI uptake and retention (Barkan et al., 2016). A comparison of demographic details did not find any factors that significantly predicted a participant’s likelihood to drop out of the study (Table 13).

Future studies on IECO should focus on creating a more robust study design to combat these limitations. A future study can reduce bias by: (1) incorporating a randomized-controlled trial design, considered the gold standard for establishing causal relationships and (2) complementing survey questionnaires with objective neurophysiological data such as EEG, fMRI, biological samples, and wearable devices. Some parameters of interest that may validate self-reported measures may include the following: cortisol measured in the bodily fluids or hair to indicate stress levels (Wright et al., 2015), frontal midline theta rhythm measured by EEG to indicate attentional awareness (Ishii et al., 1999), anatomical changes in the brain measured by fMRI, or changes in heart rate variability or sleep architecture measured by wearable devices (Maruthai et al., 2016). To combat attrition rate, future studies could incorporate greater incentives for participants to complete all the study timepoints. This could include compensating participants for their time and using a more comprehensive system for study follow-up communication. In addition, due to limitations of the COVID-19 pandemic, all study procedures were completed virtually. Studies that require participants to commit to in-person study visits may be more effective in retaining participants.

## Conclusion

This study provides comprehensive evidence that accessible mind–body interventions such as Shambhavi Mahamudra Kriya taught through the fully virtual Inner Engineering Completion Online program can result in sustained reductions in stress and improvements in overall well-being. Longitudinal data collected through validated scales on stress, mindfulness, and well-being, shows that improvements resulting from the practice of Shambhavi Mahamudra Kriya are significant and long-lasting.

## Data Availability

The raw data supporting the conclusions of this article will be made available by the authors, without undue reservation.
